# miR-219a-5p enhances the radiosensitivity of non-small cell lung cancer cells through targeting CD164

**DOI:** 10.1042/BSR20192795

**Published:** 2020-07-23

**Authors:** Tao Wei, Shan Cheng, Xiao Na Fu, Lian Jie Feng

**Affiliations:** 1 Department of Radiation, Anyang Tumor Hospital, The Fourth Affiliated Hospital of Henan University of Science and Technology, Anyang 455000, China; 2 Department of Histology and Embryology, Xinxiang Medical University, Xinxiang 453000, China

**Keywords:** apoptosis, CD164, miR-219a-5p, non-small cell lung carcinoma, radiosensitivity

## Abstract

Lung cancer is one of the leading causes of cancer-associated mortality. Non-small cell lung carcinoma (NSCLC) accounts for 70–85% of the total cases of lung cancer. Radioresistance frequently develops in NSCLC in the middle and later stages of radiotherapy. We investigated the role of miR-219a-5p in radioresistance of NSCLC. miR-219a-5p expression in serum and lung tissue of lung cancer patients was lower than that in control. Compared with radiosensitive (RS) NSCLC patients, miR-219a-5p expression was decreased in serum and lung tissue in radioresistant patients. miR-219a-5p expression level was negatively associated with radioresistance in NSCLC cell lines. Up-regulation of miR-219a-5p increased radiosensitivity in radioresistant NSCLC cells *in vitro* and *in vivo*. Down-regulation of miR-219a-5p decreased radiosensitivity in radiosensitive A549 and H358 cells. miR-219a-5p could directly bind in the 3′UTR of CD164 and negatively regulated CD164 expression. CD164 expression was higher in radioresistant NSCLC tissues than RS tissues. Up-regulation of CD164 significantly inhibited miR-219a-5p-induced regulation of RS in radioresistant A549 and H358 cells. Down-regulation of CD164 significantly inhibited the effect of anti-miR-219a-5p on radiosensitive A549 and H358 cells. miR-219a-5p or down-regulation of CD164 could increase apoptosis and γ-H2A histone family member X (γ-H2AX) expression in radioresistant cells *in vitro* and *in vivo*. Up-regulation of CD164 could inhibit the effect of miR-219a-5p on apoptosis and γ-H2AX expression. Our results indicated that miR-219a-5p could inhibit CD164, promote DNA damage and apoptosis and enhance irradiation-induced cytotoxicity. The data highlight miR-219a-5p/CD164 pathway in the regulation of radiosensitivity in NSCLC and provide novel targets for potential intervention.

## Introduction

Lung cancer is the most frequently diagnosed malignant cancer globally and one of the leading causes of cancer-associated mortality [1]. According to the statistics, the incidence of lung cancer ranks only second to prostate cancer in all malignant tumors in men and only less than breast cancer in women [2]. Lung cancer mainly consists of small cell lung carcinoma (SCLC) and non-small cell lung carcinoma (NSCLC), and NSCLC accounts for 70–85% of the total cases of lung cancer [3]. Radiotherapy combined with surgery and chemotherapy is an effective treatment for malignant tumors, including lung cancer [4].

Radiotherapy mainly indicates ionizing radiation which can induce damage to cellular DNA and subsequently result in cell death via direct or indirect mechanisms [5]. The efficacy of radiotherapy relies on the sensitivity of tumor cells to radiation. Thus, radioresistance usually occurs when tumor cells become insensitive to radiation, leading to the recovery of damaged cells [6]. In particular, radioresistance frequently develops in NSCLC in the middle and later stages of radiotherapy [7]. Thereof, it is of importance to identify new mechanisms of radioresistance in NSCLC.

In recent years, emerging studies indicate that dysregulation of microRNAs (miRNAs) is associated with radioresistance of many types of cancers [8–11]. It is demonstrated that miRNAs could not only regulate key biochemical processes that are critical for tumorigenesis [12] but also respond to radiation and regulate DNA damage response [13]. miR-219 is down-regulated in NSCLC and could inhibit tumor cell growth and metastasis [14]. In the present study, we aimed to investigate whether the dysregulation of miR-219a-5p is involved in radioresistance of NSCLC. Using tumor cell and xenograft animal models, we explored the mechanism of miR-219a-5p-induced regulation of radiosensitivity in NSCLC.

## Materials and methods

### Tissue specimens

Fresh NSCLC tissue specimens and blood samples were collected from 30 patients, who received surgical treatment at the Anyang Tumor Hospital between April 2016 and June 2017. None of the patients had received prior neoadjuvant treatment. The patients at stage II or III underwent curative resection, followed by radiotherapy (total cumulative dose, 60 Gy with photon beams of cobalt 60 and weekly dose was 10 Gy). The clinicopathological features are shown in Table 1. Radiotherapy sensitivity was mainly assessed according to the overall survival rate and local or distant recurrence rate. Cancer growth in the same lung or in the mediastinum was defined as local recurrence. Distant recurrence was defined as a new peripheral contralateral lesion. Based on these criteria, the patients were divided into radiotherapy-sensitive (12, 40%) and -resistant (18, 60%) groups. Thirteen non-tumor lung tissues and blood samples were collected from non-tumor patient with thoracic trauma. Tissue samples were immediately frozen in liquid nitrogen and stored at −80°C. Serum samples were extracted from whole blood through centrifugation at 2800×***g*** for 10 min and stored at −80°C. Written informed consent was obtained from all patients. The protocol was approved by the Ethics Committee of Anyang Tumor Hospital.

**Table 1:** Clinicopathological characteristics of the included patients

	Number	Sensitive group	Resistant group	*P*-value
**Gender**				
Male	13	4	9	
Female	17	8	9	0.451
**Age (years)**				
≥60	21	7	14	
<60	9	5	4	0.427
**Surgical procedure**				
Single lobectomy	16	5	11	
Bilobectomy	6	1	5	
Pneumonectomy	8	6	2	0.638
**TNM classification**				
T1	0	-	-	
T2	11	7	4	
T3	19	5	14	0.236
N				
N0	0	-	-	
N1	5	1	4	
N2	25	11	14	0.425
**Stage**				
I	0	-	-	
II	10	4	6	
III	20	8	12	0.218
**Histologic type**				
Squamous cell	22	10	12	
Adenocarcinoma	5	1	4	
Large cell and others	3	1	2	0.562

TNM: Tumor-Node-Metastasis.

### Cell culture

NSCLC cell lines, A549, H23, H358, H520, H460, H841 and H1299, were cultured in RPMI 1640 (Life Technologies, Carlsbad, CA, U.S.A.) supplemented with heat-inactivated 10% FBS (Life Technologies, Carlsbad, CA, U.S.A.), 1% Penicillin/Streptomycin (P/S, Life Technologies, Carlsbad, CA, U.S.A.), and cultured at 5% CO_2_ and 37°C.

### Irradiation treatment

For the induction of irradiation, a Gammacell® 40 Exactor (Atomic Energy of Canada Limited, Chalk River, ON, Canada) was employed. ^137^Cs γ-ray irradiation was delivered at a dose rate of 1 Gy/min. The cells were exposed to 0–10 Gy irradiation for the observational experiments. Then, 5 Gy irradiation was used for the subsequent experiments. A549 and H358 cells were exposed to sequentially increasing doses of irradiation to generate radioresistant NSCLC cell lines. The established radioresistant cell lines were defined as A549-(radioresistant) RR and H358-RR cells. The parental radiosensitive cell lines were defined as A549-(radiosensitive) RS and H358-RS cells.

### Transfection of miRNAs and plasmids

To evaluate the function of miR-219-5p, cells were transfected with miR-219-5p inhibitor, miR-219-5p mimics or miR-NC (negative control) (Thermo Fisher Scientific, Inc., U.S.A.). To evaluate the function of CD164, cells were transfected with pcDNA3.1 (Invitrogen; Thermo Fisher Scientific, Inc., U.S.A.)-CD164, shCD164 or NC (Santa Cruz, CA, U.S.A.). The transfection was performed using Lipofectamine® 2000 and Opti-MEM I reduced serum medium (Invitrogen; Thermo Fisher Scientific, Inc., U.S.A.), as per the manufacturer’s instructions. Forty-eight hours post transfection, cells were used for further examination.

### Luciferase assay

HEK293 cells were co-transfected with 100 nM miR-219-5p inhibitor, miR-219-5p mimics or miR-NC, 250 ng pGL3 reporter vector carrying CD164-3′UTR with WT or mutant miR-219-5p binding site, 25 ng of the phRL-SV40 control vector (Promega, Madison, WI, U.S.A.) in 24-well plates. Twenty-four hours post-transfection, firefly luciferase activity was measured using a Dual Luciferase Assay Kit (Promega, Madison, WI, U.S.A.). The normalization to *Renilla* luciferase reference plasmid was performed to evaluate the relative reporter gene activity.

### Cell viability

At the end of each experiment, the culture medium was replaced with fresh serum-free RPMI 1640 medium. Ten microliters of CCK8 solution was added into a well of 96-well plates. The plates were placed in the incubator for 1–2 h. Finally, the absorbance at 460 nm was measured by the use of an ELISA reader. The results were shown as fold-change of control.

### Evaluation of apoptosis

Apoptosis was measured using a TUNEL staining assay kit (Roche, Basel, Switzerland) according to the manufacturer’s protocols. The percentage of apoptotic cells were analyzed by flow cytometry (BD, C6, U.S.A.). Relative apoptosis was expressed as percentage of control. In transplanted tumor tissues, mRNA and protein levels of Bax and Bcl-2 were determined to evaluate apoptosis.

### Real-time PCR

Isolation of total RNA from cells and tissues was performed using TRIzol reagent (Invitrogen, U.S.A.) according to the instructions. cDNA was synthesized using a Revert-Aid First Strand cDNA Synthesis Kit (Thermo Scientific, U.S.A.) according to the manufacturer’s protocols. Real-time qPCR was conducted on a Bio-Rad CFX96 Detection System (Bio-Rad, U.S.A.) using the SYBR Green Master kit (Takara, China). For the measurement of miR-219a-5p level, total RNA was polyadenylated by poly (A) polymerase (Ambion, Austin, TX, U.S.A.) according to the manufacturer’s instructions. Reverse transcription was performed using an ImPro-II Reverse Transcriptase (Promega, Madison, WI, U.S.A.), according to the manufacturer’s instructions. β-actin and U6 were used as internal controls. 2^−ΔΔ*C*_t_^ method was used to evaluate the relative level of mRNA.

### Western blot

Total protein was extracted from cell lines and tumor tissues using RIPA lysis buffer supplemented with protease inhibitors (1 mM phenylmethylsulfonyl fluoride, 1 μg/ml aprotinin, 1 μg/ml leupeptin and 1 mM Na_3_VO_4_). After the quantification of protein by BCA method, the samples were separated by SDS/PAGE and transferred on to a PVDF membrane. The membranes were blocked with 5% BSA for 1 h at room temperature. Then the membranes were incubated with specific primary antibodies (γH2A histone family member X (γH2AX); Santa Cruz Biotechnology, 1:500), CD164 (Thermo Fisher Scientific, 1:1000), β-actin (Sigma–Aldrich, 1:1000)) overnight at 4°C. After washing with TBST, the membranes were incubated with HRP–conjugated secondary antibodies at 37°C for 1 h. Protein bands were visualized using enhanced chemiluminescence reagent (Thermo Fisher Scientific, U.S.A.).

### Xenograft experiments

All animal experiments were approved by Institutional Animal Care and Use Committee of Anyang Tumor Hospital. Twenty-four male BALB/c nude mice (4–6 weeks) were obtained from Charles River Laboratories (Beijing, China) and housed in a certified, specific pathogen-free level animal facility. The animal experiment was performed in Anyang Tumor Hospital. A total of 4 × 10^6^ A549 radioresistant cells transfected with miR-219a-5p and pcDNA-CD164 were injected into the mouse mammary fat pads to develop xenograft tumors. The irradiation treatment was performed as previously reported [15]. After the experiment, animals were anesthetized using 2% isoflurane and killed by cervical dislocation. Tissues were then collected for further determination.

### Statistical analysis

Data were expressed as mean ± S.D. Each *in vitro* experiment was repeated at least three times. Statistical analysis was performed using GraphPad Prism 6.0 software. Differences were analyzed using a one-way analysis-of-variance (ANOVA) test followed by Tukey’s post hoc test. *P*<0.05 was considered to be statistically significant.

## Results

### miR-219a-5p was positively associated with the radiosensitivity in NSCLC

**Figure 1:**
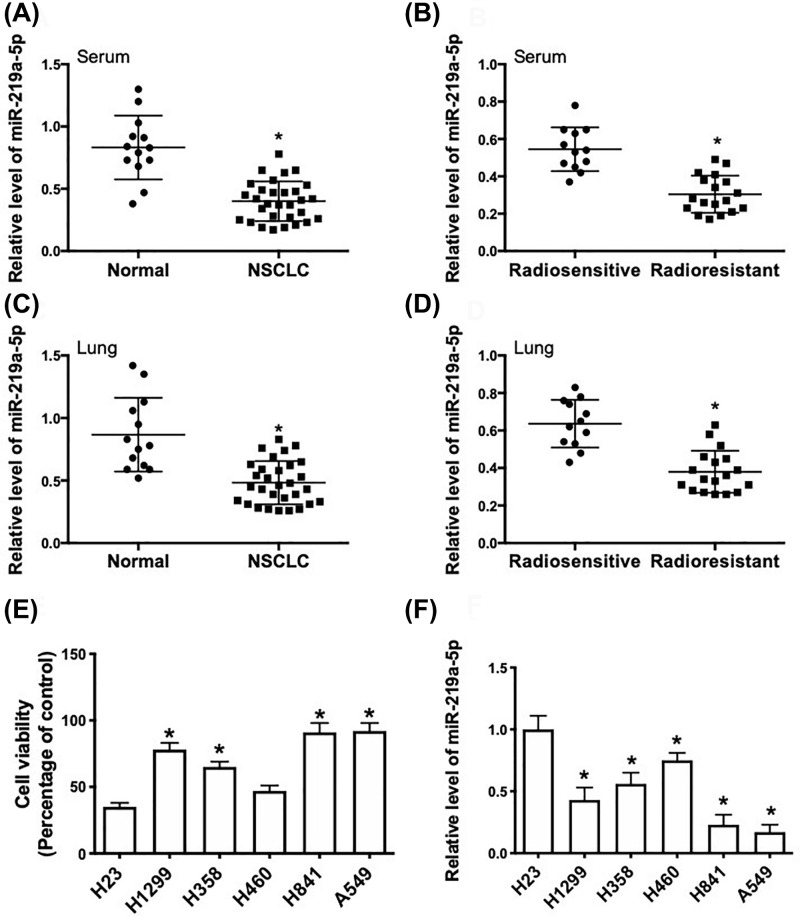
miR-219a-5p expression is negatively associated with radioresistance in lung cancer (**A**) Real-time qPCR analysis of relative miR-219a-5p expression in serum of lung cancer patients (*n*=30) and healthy controls (*n*=13). *P<0.05. (**B**) Real-time qPCR analysis of relative miR-219a-5p expression in serum of radiosensitive patients (*n*=12) and radioresistant patients (*n*=18). * *P*<0.05. (**C**) Real-time qPCR analysis of relative miR-219a-5p expression in lung tissue of lung cancer patients (*n*=30) and healthy controls (*n*=13). *P<0.05. (**D**) Real-time qPCR analysis of relative miR-219a-5p expression in lung tissue of radiosensitive patients (*n*=12) and radioresistant patients (*n*=18). *P<0.05. (**E**) NSCLC cell lines were exposed to 5 Gy of γ-ray irradiation and cell viability was determined using CCK8 assay (relative percent change of respective control). (**F**) NSCLC cell lines were exposed to 5 Gy of γ-ray irradiation and relative miR-219a-5p expression was determined using real-time qPCR (relative expression compared with H23 cells). *P<0.05, compared with H23 cells.

To investigate the potential role of miR-219a-5p in the regulation of radiosensitivity in NSCLC, we compared the expression of miR-219a-5p between NSCLC and non-tumor specimens and compared the expression of miR-219a-5p between radiosensitive and radioresistant tissues. The results showed that miR-219a-5p expression in serum and lung tissue of tumor patients was significantly lower than that in matched control samples (Figure 1A,C). In addition, miR-219a-5p expression in serum and lung tissue of radioresistant patients was significantly lower than that in radiosensitive samples (Figure 1B,D). We also determined the cell viabilities and expression levels of miR-219a-5p in a series of NSCLC cell lines after 5 Gy of γ-ray irradiation. We revealed that the expression level of miR-219a-5p was negatively correlated with the viability of NSCLC cells treated by irradiation (Figure 1E,F). The results indicated that dysregulation of miR-219a-5p expression may be associated with radioresistance in NSCLC.

### miR-219a-5p regulates radiosensitivity in NSCLC cell

**Figure 2:**
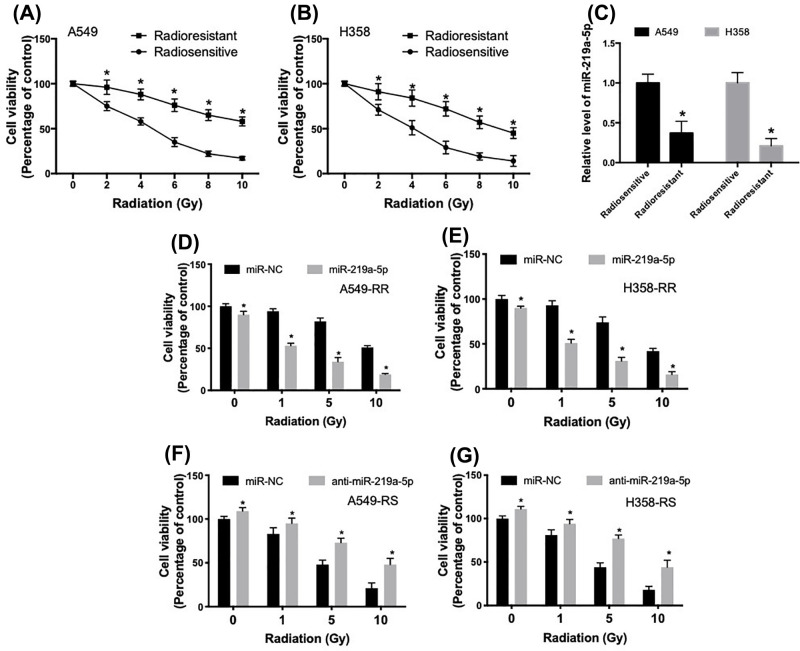
miR-219a-5p regulates radiosensitivity in NSCLC cells (**A,B**) Radioresistant NSCLC cell lines were generated using gradually increasing doses of irradiation in A549 and H358 cells. The sensitivity of radiosensitive and radioresistant A549 and H358 cells was examined using irradiation at 0, 2, 4, 6, 8 and 10 Gy, at a dose rate of 2 Gy/min. (**C**) Relative level of miR-219a-5p in A549 and H358 parental and radioresistant cells. (**D,E**) Radioresistant A549-RR and H358-RR cells were transfected with miR-219a-5p and then irradiation-induced cytotoxicity was determined using CCK8 assay. (**F,G**) Parental radiosensitive A549-RS and H358-RS cells were transfected with anti-miR-219a-5p and then irradiation-induced cytotoxicity was determined using CCK8 assay, *P<0.05.

Radioresistant NSCLC cell lines were generated using gradually increasing doses of irradiation in A549 and H358 cells. The radioresistant cell lines were defined as A549-RR and H358-RR and the parental radiosensitive cell lines were defined as A549-RS and H358-RS. The radiosensitive and radioresistant A549 and H358 cells were exposed to 0, 2, 4, 6, 8 and 10 Gy, at a dose rate of 2 Gy/min. The results confirmed the establishment of radioresistant A549 and H358 cells (Figure 2A,B). In Figure 2C, we showed that miR-219a-5p expression was decreased in radioresistant A549-RR and H358-RR cells, compared with the parental radiosensitive cells. To evaluate the role of miR-219a-5p in the development of radioresistance in A549 and H358 cells, the radioresistant in A549-RR and H358-RR cells were transfected with miR-219a-5p mimics (Supplementary Figure S1). As shown in Figure 2D,E, transfection of miR-219a-5p significantly decreased cell viability under basal condition and reduced cell viability in response to different doses of irradiation. Up-regulation of miR-219a-5p increased irradiation-induced cytotoxicity by three- to five-folds (Figure 2F,G). In addition, radiosensitive A549-RS and H358-RS cells were transfected with anti-miR-219a-5p (Supplementary Figure S2). The results showed that inhibition of miR-219a-5p in A549-RS and H358-RS cells significantly increased basal cell viability and increased cell viability after different doses of irradiation treatment. The results indicated that miR-219a-5p could regulate radiosensitivity in NSCLC cells.

### miR-219a-5p enhances radiosensitivity through inhibition of CD164 via direct targeting its 3′UTR

**Figure 3:**
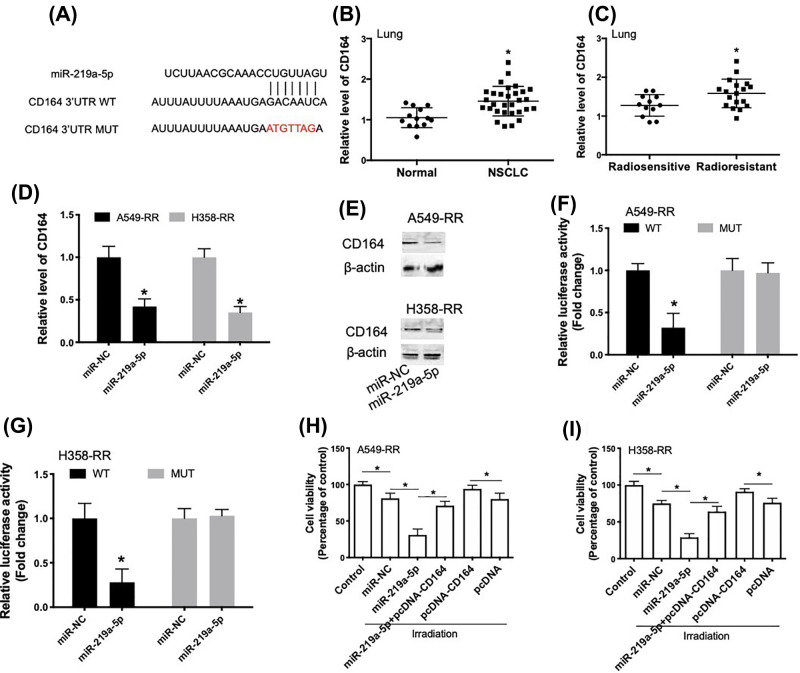
miR-219a-5p enhances radiosensitivity through inhibition of CD164 via direct targeting its 3′UTR (**A**) Putative binding site of miR-219a-5p in 3′UTR of CD164 mRNA by prediction and generated mutant site at the CD164 3′UTR seed region binding to miR-219a-5p. (**B**) Real-time qPCR analysis of relative CD164 expression in lung tissue of lung cancer patients (*n*=30) and healthy controls (*n*=13). (**C**) Real-time qPCR analysis of relative CD164 expression in lung tissues of radiosensitive patients (*n*=12) and radioresistant patients (*n*=18). (**D,E**) A549-RR and H358-RR cells were transfected with miR-219a-5p and then relative mRNA and protein expression of CD164 were determined using real-time qPCR and Western blot. (**F,G**) The effect of miR-219a-5p on reporters of CD164-wt and CD164-mut in A549-RR or H358-RR cells was measured by luciferase reporter gene assay. (**H,I**) A549-RR or H358-RR cells were transfected with miR-219a-5p in combination with or without pcDNA-CD164. The cytotoxicity of 5-Gy irradiation was determined using CCK8 assay, *P<0.05.

To investigate the mechanism of miR-219a-5p-induced regulation of radiosensitivity of NSCLC cells, we performed informatics analysis. We found that there was a putative binding site of miR-219a-5p in the 3′UTR of CD164 (Figure 3A). In addition, CD164 expression in lung tissue of tumor patients was significantly higher than that in matched control samples (Figure 3B). CD164 expression in lung tissues of radioresistant patients was significantly lower than that in radiosensitive samples (Figure 3C). Transfection of miR-219a-5p in radioresistant A549-RR and H358-RR cells induced a significant decrease in CD164 mRNA (Figure 3D) and protein (Figure 3E) expression. Luciferase reporter assay showed that in radioresistant A549-RR and H358-RR cells, miR-219a-5p could decrease CD164-WT luciferase activity but not CD164-MUT luciferase activity (Figure 3F,G). The results indicated that miR-219a-5p directly regulated CD164 mRNA level. To investigate the possible role of CD164 in miR-219a-5p-induced regulation of radiosensitivity in A549 and H358 cells, A549-RR and H358-RR cells were co-transfected with miR-219a-5p and pcDNA-CD164 (Supplementary Figure S3). We showed that miR-219a-5p-induced decrease in cell viability in irradiation-treated cells was inhibited by pcDNA-CD164 (Figure 3H,I). In addition, overexpression of CD164 inhibited irradiation-induced decrease in cell viability in A549-RR and H358-RR cells (Figure 3H,I). These results suggested that miR-219a-5p enhanced radiosensitivity through inhibition of CD164 via directly targeting its 3′UTR.

### Inhibition of miR-219a-5p promotes radioresistance through regulation of CD164

**Figure 4:**
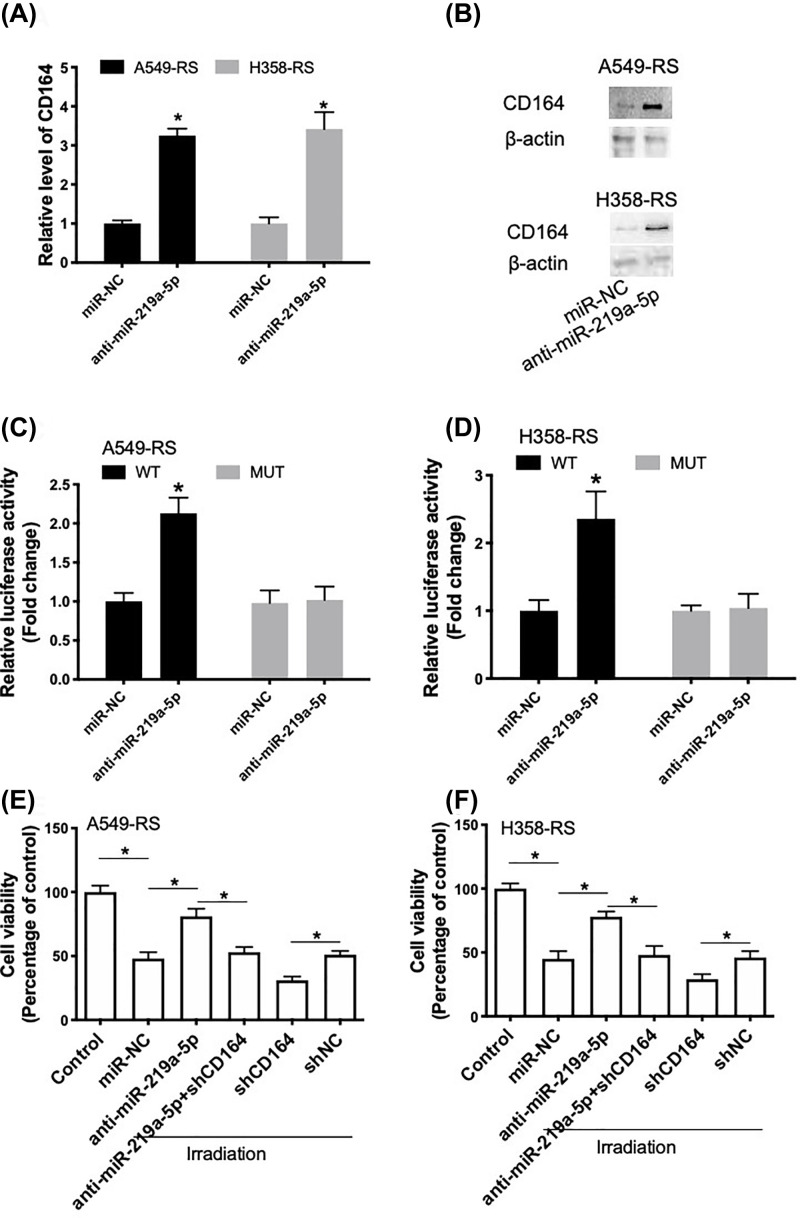
Anti-miR-219a-5p promotes radioresistance through regulation of CD164 (**A,B**) A549-RS and H358-RS cells were transfected with anti-miR-219a-5p and then relative mRNA and protein expression of CD164 were determined using real-time qPCR (A) and Western blot (B). (**C,D**) The effect of anti-miR-219a-5p on reporters of CD164-wt and CD164-mut in A549-RS or H358-RS cells was measured by luciferase reporter gene assay. (**E,F**) A549-RS or H358-RS cells were transfected with anti-miR-219a-5p in combination with or without shCD164. The cytotoxicity of 5 Gy irradiation was determined using CCK8 assay. *P<0.05.

Transfection of anti-miR-219a-5p in radiosensitive A549-RS and H358-RS cells induced a significant increase in CD164 mRNA (Figure 4A) and protein (Figure 4B) levels. Luciferase reporter assay showed that in radiosensitive A549-RS and H358-RS cells, anti-miR-219a-5p could increase CD164-WT luciferase activity but not CD164-MUT luciferase activity (Figure 4C,D). Radiosensitive A549-RS and H358-RS cells were also co-transfected with anti-miR-219a-5p and shCD164 (Supplementary Figure S4). We showed that anti-miR-219a-5p-induced increase in cell viability in irradiation-treated cells was inhibited by shCD164 (Figure 4E,F). In addition, down-regulation of CD164 promoted irradiation-induced decrease in cell viability (Figure 4E,F). These results suggested that inhibition of miR-219a-5p promoted radioresistance in NSCLC cells through regulation of CD164.

### miR-219a-5p increases the radiosensitivity of NSCLC cells through targeting CD164 *in vivo*

**Figure 5:**
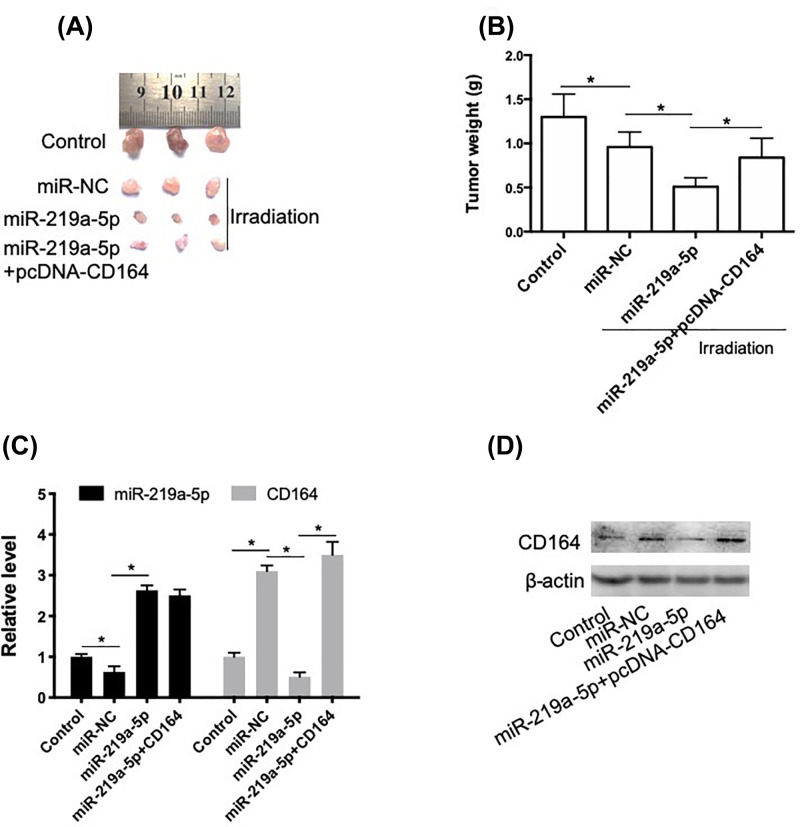
miR-219a-5p increases the radiosensitivity of NSCLC cells through targeting CD164 *in vivo* A549-RR cells with miR-219a-5p in combination with or without pcDNA-CD164. Xenograft mice were established using transplantation. (**A**) Images of dissected tumors from nude mice. (**B**) Weight of resected xenograft tumors in nude mice. (**C**) Relative expression of miR-219a-5p and CD164 was determined using real-time PCR. (**D**) Protein level of CD164 was determined using Western blot, *P<0.05.

To evaluate the role of miR-219a-5p in the regulation of radiosensitivity of NSCLC *in vivo*, we established xenograft tumor mice using transfected radioresistant A549-RR cells. As shown in Figure 5A,B, irradiation decreased the tumor size and weight in a degree. In the miR-219a-5p transfection group, the tumor size and weight significantly decreased after irradiation, compared with mice treated by irradiation alone. In addition, transfection of pcDNA-CD164 significantly inhibited the effect of miR-219a-5p on tumor growth. In Figure 5C,D, we showed that irradiation resulted in a decrease in miR-219a-5p expression and increase in CD164 mRNA and protein levels. miR-219a-5p significantly increased the mRNA and protein levels of CD164 in tumors. These results indicated that miR-219a-5p increased the radiosensitivity of NSCLC cells through targeting CD164 *in vivo*.

### Apoptosis and DNA damage were involved in miR-219a-5p/CD164-induced regulation of radiosensitivity in NSCLC cells

**Figure 6:**
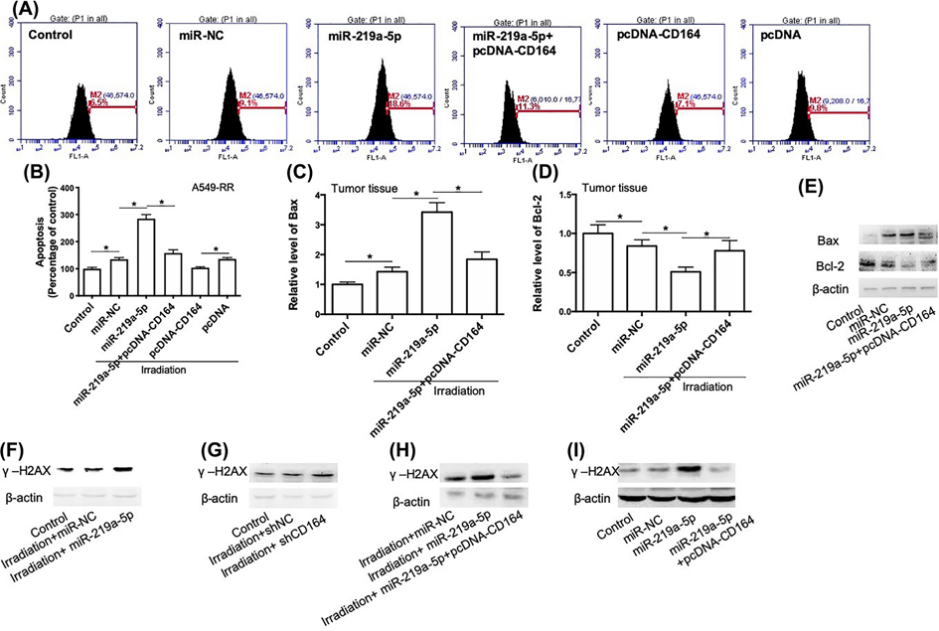
miR-219a-5p regulates radiosensitivity via CD164-mediated regulation of apoptosis and DNA damage response (**A,B**) A549-RR cells were transfected with miR-219a-5p in combination with or without pcDNA-CD164 and then exposed to 5 Gy irradiation. Apoptosis was evaluated using a TUNEL assay kit. (A) Representative flow analysis images. (B) Statistical results. (**C**–**E**) mRNA and protein levels of Bax and Bcl-2 in xenograft tumor in mice were determined using real-time qPCR and Western blot. (**F**) A549-RR cells were transfected with miR-219a-5p and then exposed to 5 Gy irradiation. Protein expression of γ-H2AX was determined by Western blot. (**G**) A549-RR cells were transfected with shCD164 and then exposed to 5 Gy irradiation. Protein expression of γ-H2AX was determined by Western blot. (**H**) A549-RR cells were transfected with miR-219a-5p in combination with or without pcDNA-CD164 and then exposed to 5 Gy irradiation. Protein expression of γ-H2AX was determined by Western blot. (**I**) Protein expression of γ-H2AX in xenograft tumor in mice was determined by Western blot. *P<0.05.

To explore the mechanism of miR-219a-5p/CD164-induced regulation of radiosensitivity in NSCLC cells, we determined the role of miR-219a-5p and CD164 in the regulation of apoptosis in radioresistant A549-RR and H358-RR cells. As shown in Figure 6A,B, miR-219a-5p transfection significantly increased irradiation-induced apoptosis in radioresistant A549-RR and H358-RR cells. Up-regulation of CD164 repressed miR-219a-5p-induced effect on apoptosis (Figure 6A,B). Overexpression of CD164 could also decrease apoptosis induced by irradiation in radioresistant A549-RR and H358-RR cells (Figure 6A,B). In xenograft tumor tissues, irradiation-induced increase in Bax expression and decrease in Bcl-2 expression were enhanced by miR-219a-5p (Figure 6C–E). This effect of miR-219a-5p was inhibited by CD164 (Figure 6C–E). Moreover, we also determined the effect of up-regulation of miR-219a-5p and CD164 on the expression of γ-H2A histone family member X (γ-H2AX). We found that irradiation had little effect on γ-H2AX expression in both cells and tumor tissues (Figure 6F–I). In response to irradiation, γ-H2AX expression could be significantly increased by either up-regulation of miR-219a-5p or down-regulation of CD164 (Figure 6F–I). Furthermore, miR-219a-5p-induced increase in γ-H2AX expression was significantly inhibited by up-regulation of CD164 (Figure 6F–I). These results demonstrated that apoptosis and DNA damage were involved in miR-219a-5p/CD164-induced regulation of radiosensitivity in NSCLC cells.

## Discussion

Radioresistance is a major obstacle in the treatment of NSCLC [16]. Emerging evidence supports the notion that dysregulation of miRNAs plays an important role in the occurrence of radioresistance in NSCLC [17]. However, the mechanism of miRNAs dysregulation-induced radioresistance in NSCLC is far from being completely understood. In recent years, abnormal expression of miR-219 is associated with the development of various types of tumors. It has been shown that miR-219 plays a tumor suppressive role in glioma [18], malignant melanoma [19], esophageal squamous cell carcinoma [20], colorectal cancer [14] and epithelial ovarian cancer [21]. In particular, miR-219 level is down-regulated in NSCLC and inhibits cell growth and metastasis [22]. However, there is no literature reporting the possible role of miR-219 in radioresistance of NSCLC.

In the present study, we verified the expression pattern of miR-219a-5p in patients and identified that miR-219a-5p levels in serum and lung tissues in tumor patients were increased, compared with matched control samples. Moreover, miR-219a-5p levels in serum and lung tissues in radioresistant patients were higher than that in radiosensitive patients. We also used cells and xenograft tumor mice to verify the role of miR-219a-5p. We revealed that up-regulation of miR-219a-5p significantly increased the radiosensitivity in radioresistant NSCLC cells *in vitro* and *in vivo*. Down-regulation of miR-219a-5p notably decreased the radiosensitivity in radiosensitive NSCLC cells. The results suggest that, in addition to previously reported tumor-suppressive role, miR-219a-5p also plays a role in promoting radiosensitivity in NSCLC.

A previous study has shown that miR-219 inhibits the proliferation, migration and invasion of medulloblastoma cells by targeting CD164 [23]. It is also shown that inhibition of CD164 expression in colon cancer cell line HCT116 reduces cancer cell proliferation, mobility, and metastasis *in vitro* and *in vivo* [24]. CD164 could promote tumor progression and predict the poor prognosis of bladder cancer [25]. CD164 has also been found to promote lung tumor-initiating cells with stem cell activity and determine tumor growth and drug resistance via Akt/mTOR signaling [26]. These results indicate that CD164 plays a tumor-promoting role. In the present study, we found that CD164 expression was higher in radioresistant patients, compared with that in radiosensitive patients. Overexpression of CD164 significantly inhibited miR-219a-5p-induced increase in radiosensitivity in NSCLC cells *in vitro* and *in vivo*. miR-219a-5p could also directly regulate the mRNA 3′UTR of CD164. The results suggested that miR-219a-5p could regulate radiosensitivity in NSCLC via directly regulating CD164 expression.

Apoptosis is an important mechanism of irradiation-induced cytotoxicity [27]. In the current study, we also examined the role of miR-219a-5p and CD164 in the regulation of apoptosis in the context of irradiation. Our results showed that miR-219a-5p significantly enhanced irradiation-induced apoptosis *in vitro* and *in vivo*. CD164 played an inhibitory effect on apoptosis in the context of irradiation. Overexpression of CD164 could inhibit miR-219a-5p-induced enhancement of apoptosis in response to irradiation. Moreover, we evaluated the role of miR-219a-5p and CD164 in the regulation of DNA damage in irradiation-treated NSCLC cells. Our results showed that the DNA damage marker γ-H2AX was increased by up-regulation of miR-219a-5p and down-regulation of CD164. Up-regulation of CD164 could inhibit miR-219a-5p-induced up-regulation of γ-H2AX in irradiation-treated cells. The results demonstrated that apoptosis and DNA damage may be important mechanisms underlying miR-219a-5p/CD164-induced regulation of radiosensitivity in NSCLC cells.

**Figure 7:**
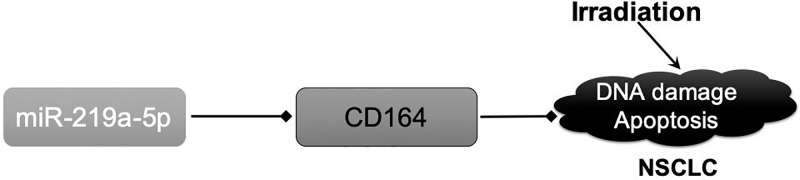
Mechanistic figure of miR-219a-5p-induced regulation of radiosensitivity in NSCLC via regulation of CD164

In summary, our results revealed that miR-219a-5p enhanced radiosensitivity in NSCLC cells *in vitro* and *in vivo*. miR-219a-5p could inhibit CD164, promote DNA damage and apoptosis and enhance irradiation-induced cytotoxicity (Figure 7). Our data highlight the importance of miR-219a-5p/CD164 pathway in the regulation of radiosensitivity in NSCLC and provide novel targets for potential intervention.

## Supplementary Material

Supplementary Figures S1-S4

## Data Availability

The data will be available on reasonable request.

## Abbreviations

Bax: BCL2-associated X
Bcl-2: B-cell lymphoma-2
CCK-8: cell counting kit-8
HRP: horseradish peroxidase
miRNA: microRNA
NC: negative control
NSCLC: non-small cell lung carcinoma
qPCR: quantitative ploymerase chain reaction
RIPA: radio immunoprecipitation assay
TUNEL: terminal deoxynucleotidyl transferase-mediated dUTP nick-end labeling
γ-H2AX: γ-H2A histone family member X
