# Supplementary material for: miR-219a-5p enhances the radiosensitivity of non-small cell lung cancer cells through targeting CD164
Source: Biosci Rep. 2020 Jul 23;40(7):BSR20192795. doi: 10.1042/BSR20192795 (PMC7378263; doi:10.1042/BSR20192795)
Supplement: Supplementary Figures S1-S4 [file BSR-2019-2795_supp.pdf]

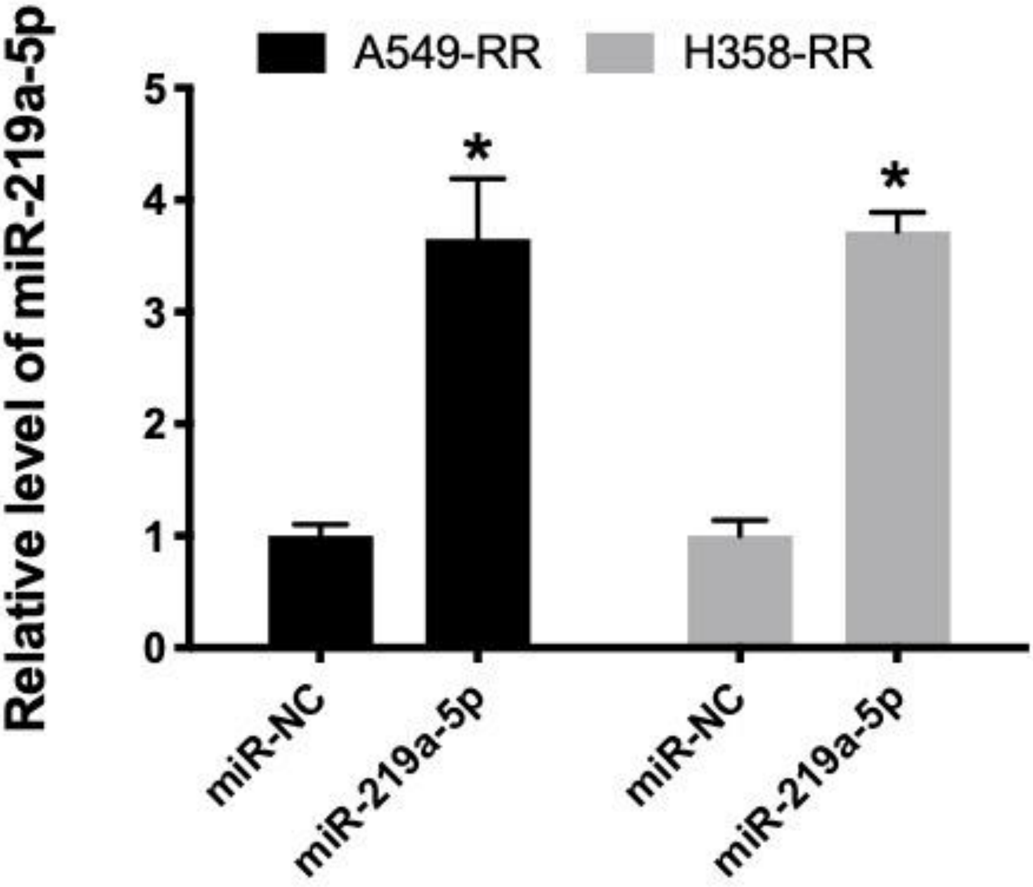

SFig. 1. Transfection efficiency of miR-219a-5p in radioresistant A549-RR and H358-RR cells.

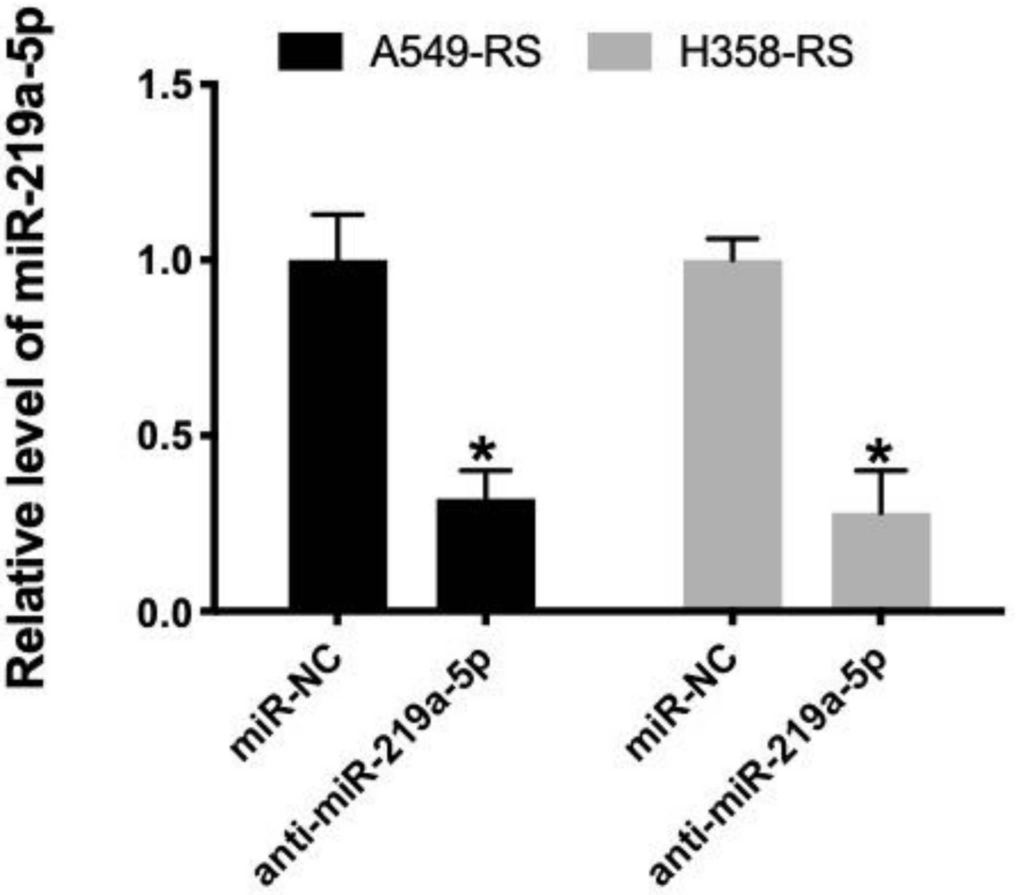

SFig. 2. Transfection efficiency of anti-miR-219a-5p in radiosensitive A549-RS and H358-RS cells.

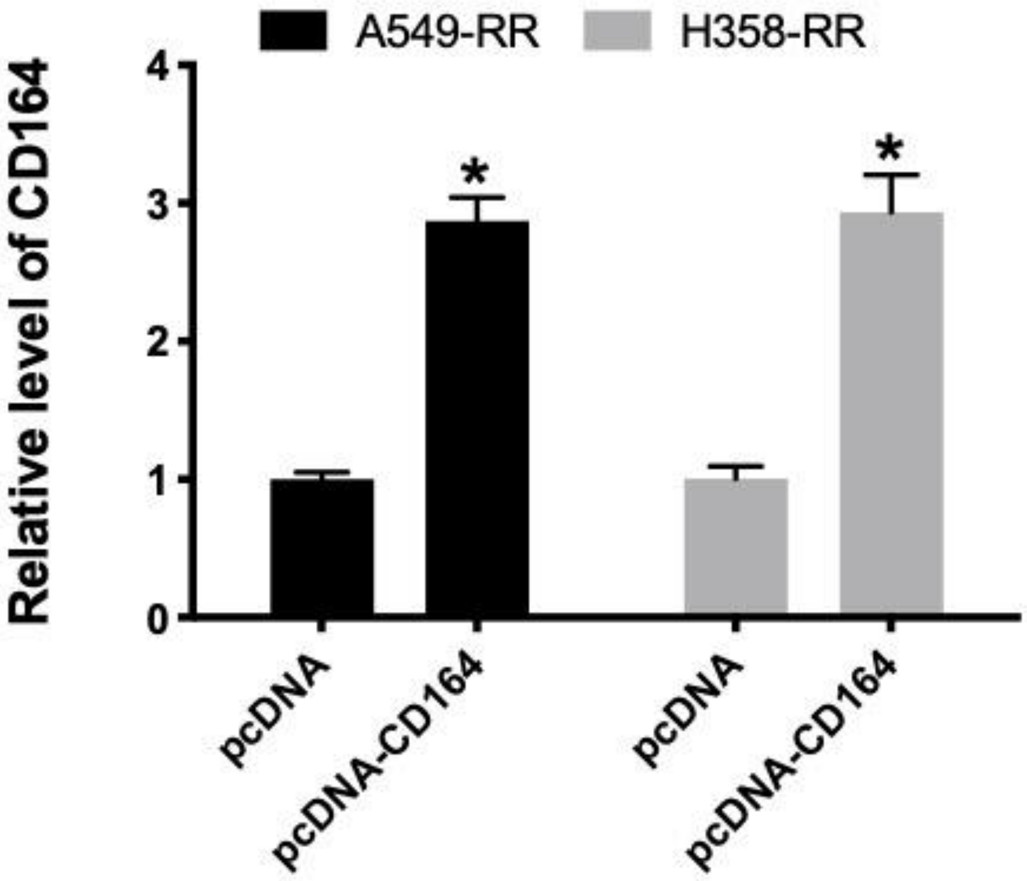

SFig. 3. Transfection efficiency of pcDNA-CD164 in radioresistant A549-RR and H358-RR cells.

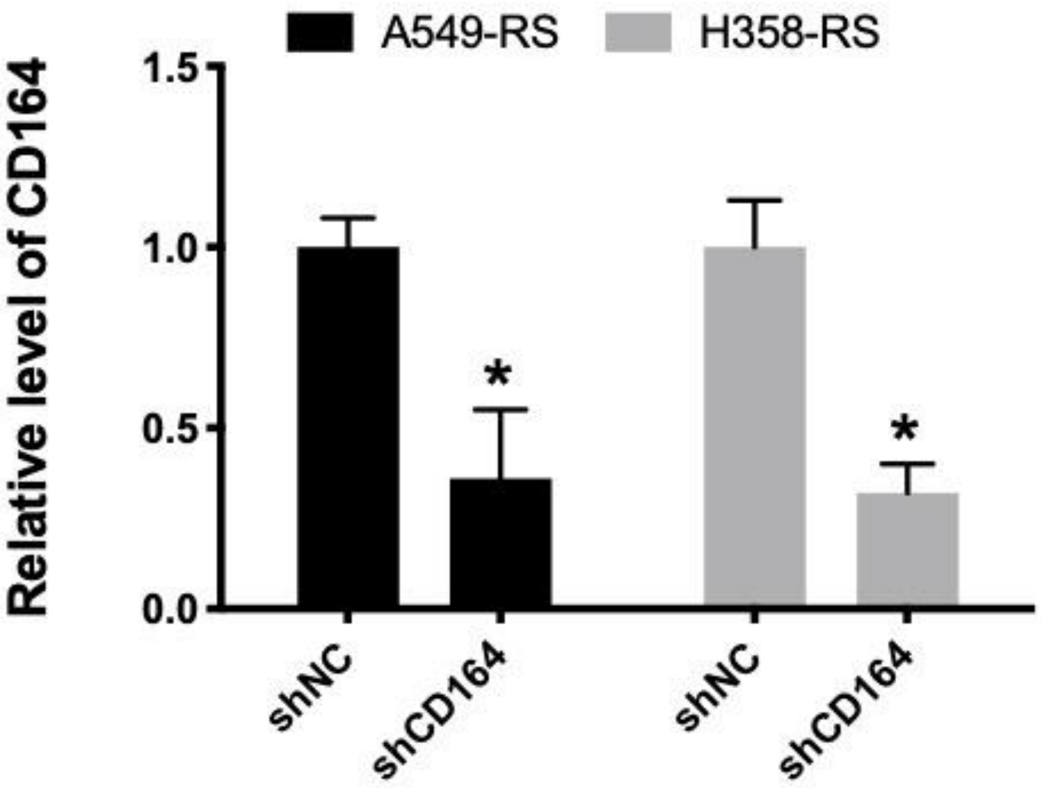

SFig. 4. Transfection efficiency of shCD164 in radiosensitive A549-RS and H358-RS cells.
